# Synergy of GSK-J4 With Doxorubicin in KRAS-Mutant Anaplastic Thyroid Cancer

**DOI:** 10.3389/fphar.2020.00632

**Published:** 2020-05-13

**Authors:** Bo Lin, Bing Lu, I-yun Hsieh, Zhen Liang, Zicheng Sun, Yang Yi, Weiming Lv, Wei Zhao, Jie Li

**Affiliations:** ^1^ Department of Breast and Thyroid Surgery, The First Affiliated Hospital of Sun Yat-sen University, Guangzhou, China; ^2^ Institute of Urology of Shenzhen University, The Third Affiliated Hospital of Shenzhen University, Shenzhen Luohu Hospital Group, Shenzhen, China; ^3^ Department of Breast Surgery, Guangzhou Women and Children’s Medical Center, Guangzhou, China; ^4^ Key Laboratory of Stem Cells and Tissue Engineering (Sun Yat-sen University), Ministry of Education, Guangzhou, China; ^5^ RNA Biomedical Institute, Sun Yat-sen Memorial Hospital, Sun Yat-sen University, Guangzhou, China; ^6^ Department of Research and Development, Shenzhen Institute for Innovation and Translational Medicine, Shenzhen, China

**Keywords:** anaplastic thyroid cancer, epigenetics, GSK-J4, synergistic action, KRAS-mutant

## Abstract

**Background:**

Anaplastic thyroid cancer is the most aggressive thyroid cancer and has a poor prognosis. At present, there is no effective treatment for it.

**Methods:**

Here, we used different concentrations of GSK-J4 or a combination of GSK-J4 and doxorubicin to treat human Cal-62, 8505C, and 8305C anaplastic thyroid cancer (ATC) cell lines. The *in vitro* experiments were performed using cell viability assays, cell cycle assays, annexin-V/PI binding assays, Transwell migration assays, and wound-healing assays. Tumor xenograft models were used to observe effects *in vivo*.

**Results:**

The half maximal inhibitory concentration (IC50) of GSK-J4 in Cal-62 cells was 1.502 μM, and as the dose of GSK-J4 increased, more ATC cells were blocked in the G2-M and S stage. The combination of GSK-J4 and doxorubicin significantly increased the inhibitory effect on proliferation, especially in KRAS-mutant ATC cells *in vivo* (inhibition rate 38.0%) and *in vitro* (suppresses rate Fa value 0.624, CI value 0.673). The invasion and migration abilities of the KRAS-mutant cell line were inhibited at a low concentration (p < 0.05).

**Conclusions:**

The combination of GSK-J4 with doxorubicin in KRAS-mutant ATC achieved tumor-suppressive effects at a low dose. The synergy of the combination of GSK-J4 and doxorubicin may make it an effective chemotherapy regimen for KRAS-mutant ATC.

## Introduction

The prognosis of anaplastic thyroid cancer (ATC) patients is poor, which have a median survival of 3–12 months (Subbiah et al., 2018; Lin et al., 2019). And their overall survival (OS) and survival rate have not been significantly improved in the past 40 years, suggesting that there is no effective treatment for improving long-term prognosis. Because of the aggressive nature and limited treatment methods, it is necessary to explore effective chemotherapies with less toxic side effects (Ezaki et al., 1992; Arora et al., 2014; Hanley et al., 2015). Combination of doxorubicin and radiation for ATC treatment was widely accepted from 1980s (Kim and Leeper, 1983; Tennvall et al., 1994; Sun et al., 2013). Despite its cardiac toxicity, doxorubicin has been the most commonly used drug in ATC treatment (Sun et al., 2013; Fan et al., 2020). Doxorubicin was considered as the most effective drug for ATC until the randomized study of the Eastern Cooperative Oncology Group (ECOG) shew the combination of cisplatin and doxorubicin was more effective than doxorubicin alone (Denaro et al., 2013). At present, the key for ATC treatment is multimodal therapy. Several studies have shown that the combination of surgery, radiation therapy with chemotherapy (such as doxorubicin), might improve the 1-year OS to more than 40%(Baek et al., 2017; Prasongsook et al., 2017; Fan et al., 2020). Targeted therapy is another possible option, especially for patients with BRAF V600E mutation. Dapafenib combined with trametinib has been proved to have clinical activity (Kapiteijn et al., 2012; Hsu et al., 2014; Jin et al., 2017; Molinaro et al., 2017; Iyer et al., 2018; Subbiah et al., 2018). Whether targeted therapy is beneficial to the long-term survival of patients with ATC has not been determined (Raue and Frank-Raue, 2016; Cao et al., 2019; Ljubas et al., 2019).

In recent years, research on histone modification affecting tumorigenesis and development has provided a target for drugs. Histone gene modification can effectively regulate gene expression levels. However, not all modification types have a stable distribution. Lysine methylation was found to be enriched in the coding region, and each methylation site corresponded to a special distribution pattern (Jin et al., 2017; Molinaro et al., 2017). These relationships provide the possibility to study the relationship between histone methylation and oncogene expression. In the present study, we knew that H3K27me3 expression was upregulated in thyroid cancer, particularly in those with a less differentiated phenotype (Tsai et al., 2019).

The methyltransferase JMJD3, which generates H3K27me3 (trimethylated lysine 27 on histone 3) alterations, consists of a JmjC catalytic domain and a C terminus, which combine to form a larger binding area. JMJD3 was found to be associated with cell proliferation and differentiation, and its expression was elevated under the stimulation of inflammation, viruses, tumors, and other factors (Lin et al., 2008; Zhu et al., 2014). Elevating its expression level in specific types of tumors may trigger an immune response and thus promote the progression of tumors. Specific inhibitors of histone methyltransferases (HMTs) with lysine and arginine residues have been developed. GSK-J4, an inhibitor of JMJD3 (Jumonji domain-containing protein 3), is one of them. GSK-J4 is an ethyl ester derivative of the H3K27 methyltransferase inhibitor GSK-J1 (Di Desidero et al., 2017). GSK-J4 can regulate the expression of downstream genes, such as NOTCH1, TNF-a, and PTEN, by inhibiting the activity of JMJD3 and affecting cell proliferation and the expression of stem cell-related genes in cancer cells (Lin et al., 2012; Arora et al., 2014; Abdulghani et al., 2016; Broecker-Preuss et al., 2016; Bible and Ryder, 2016; Park et al., 2017; Lee et al., 2018).

In this manuscript, we used GSK-J4 and doxorubicin to treat human ATC cell lines (Cal-62, 8505C, and 8305C) and found that GSK-J4 significantly inhibited the proliferation of ATC cells. The combination of GSK-J4 and doxorubicin had a stable synergistic effect on KRAS-mutant cell lines, which inhibited sphere formation, tumorigenicity, migration, and invasion of cells at a low dose of doxorubicin. GSK-J4 combined with doxorubicin may be an effective chemotherapy regimen for ATC.

## Materials and Methods

### Cell Lines

The human Cal-62, 8505C, and 8305C anaplastic thyroid cell lines used in this study were purchased from GuangZhou Jennio Biotech Co. Cal-62 is KRAS G12R mutated and BRAF wide type (WT), while 8305C and 8505C are BRAF V600E mutated. Cal-62 cells were cultured in Dulbecco’s modified Eagle’s medium (DMEM) with 1 0% fetal bovine serum (FBS), penicillin (50 units/ml; Gibco), and streptomycin (50 µg/ml; Gibco) as previously described. 8505C and 8305C cells were cultured in Minimum Essential Medium (MEM) with 10% FBS, penicillin (50 unit/ml; Gibco), and streptomycin (50 µg/ml; Gibco). All cell lines were grown at 37°C in a 5% CO_2_ atmosphere.

### CellTiter-Glo^®^ Luminescent Cell Viability Assay

Cell proliferation was measured by cell viability assays. For the cell viability assay, cells were seeded in a 96-well plate at a density of 3,000 cells/well. After recovering for 24 h, cells were treated with complete media alone or media containing GSK-J4 (maximum concentration = 20 μM, double dilution, TargetMol) or doxorubicin (maximum concentration = 10 μM, double dilution, Whiga) at different concentrations for 48 h. For the time-related cell viability assays, cells were recovered for 24 h and were then treated with complete media alone or GSK-J4 (1 µM), and cell viability was recorded every day. Cell viability was measured by using a CellTiter-Glo (CTG) mixture according to the manufacturer’s instructions. The amount of ATP was directly proportional to the number of cells present in culture. ATP was quantified by using a fluorescence microplate. Viability was calculated using a background-corrected absorbance according to the following formula: viability (%) = A of experiment well/A of control well × 100. Dose reduction index (DRI) means a multiple reduction in the dose of a drug used at a level of inhibition produced by a combination of drugs over that used alone to achieve the same level of inhibition, and the calculation formula is DRI= (Dx)_1_/(D)_1_ or (Dx)_2_/(D)_2_.

To obtain the IC50 (half maximal inhibitory concentration), GraphPad Prism 6 software was used to analyze the data. Calcusyn 2.0 software was used to calculate the combination index (CI), DRI, and Fa value.

### Transwell Migration and Invasion Assays

Transwell chambers (Guangzhou Sagene Technology Co.) with transparent PET membranes (8.0 μm pore size) were inserted into a 24-well culture plate (Corning, NY 14831, USA). For the cell invasion assay, the upper surface of the PET membrane was equally covered with 100 μl of 1.25 mg/ml Matrigel (Shanghai Pharmaceuticals Holding Co.). Briefly, 300 μl of serum-free cell suspension containing 2 × 10^5^ cancer cells was added to the upper chamber, and the cancer cells were treated with different concentrations of GSK-J4 (0, 1.25, 2.5, or 5 µM) or a combination of GSK-J4 or/and doxorubicin (DOX: GSK-J4 = 0.156:0.078 μM) at different concentrations. After incubation at 37°C in 5% CO_2_ for 24 h, the cells were fixed with methanol and stained with crystal violet staining solution. On the upper surface of the membrane, remaining nonmigrating cells were cleared with cotton swabs. The migratory and invasive cells on the lower surface of the membrane in each chamber were counted randomly under high-power fields at least five times.

The migration rate of ATC cells was determined by a wound-healing assay. The cells were grown to confluence on six-well plates. A scratch was made through the cell monolayer using a 1,000 μl pipette tip. After washing with phosphate-buffered saline (PBS) three times, maintenance medium containing 0.2% FBS was added, and the cancer cells were treated with medium, GSK-J4 or doxorubicin alone, or a combination of GSK-J4 and doxorubicin (DOX: GSK-J4 = 0.156:0.078 μM). After making the scratch, images of the wounded area were captured immediately (0-h time point). The migration of cells into the wounded area was recorded once every 4 h for 24 h using an inverted microscope (Nikon).

### Cell Cycle Analysis

After treatment with different concentrations of GSK-J4 for 48 h, the cells were harvested and resuspended in 500 μl of DNA staining solution and stained by the addition of 5 μl of permeabilization solution. After incubation at room temperature for 15 min, stained cells were immediately analyzed by flow cytometry.

### Cellular Apoptosis Assays

Both adherent cells harvested by trypsinization and floating cells collected by centrifugation were used for the annexin-V/PI binding assay. After treatment with different concentrations of GSK-J4 for 48 h, the cells were harvested, resuspended in 500 μl of 1× binding buffer, and stained by adding 2.5 μl of FITC-annexin V and 5 μl of PI working solution. When detecting synergy, after treatment with GSK-J4 or/and doxorubicin (DOX: GSK-J4 = 0.156:0.078 μM), Cal-62 cells were collected and resuspended in 500 μl of binding buffer. After 5 μl of YO-PRO-1 and 5 μl of 7-AAD were added for each well. After incubation at room temperature in the dark for 10 min, stained cells were immediately analyzed by flow cytometry.

After treatment with GSK-J4 or/and doxorubicin (DOX: GSK-J4 = 0.156:0.078 μM), RIPA buffer with protease inhibitor cocktail (Roche 4693132001) was used for lysing Cal-62 cells. Samples were diluted with 0.25 volume to 5× SDS-PAGE Sample Buffer (GenStar). For making albumen denaturation, the proteins were heated for 8 min at 100°C. SDS-PAGE was used to perform Gel electrophoresis, and then proteins were transferred to Immun-Blot PVDF Membrane (Bio-Rad). The primary antibodies used were caspase 3 (1:1000, Affinity Cat# AF6311), Pro-caspase 3 (1:1000, Abcam Cat# ab32150), and β-actin (1:3000, Proteintech Cat# 60008). Membranes were incubated with aforementioned primary antibodies for 16–20 h at 4°C, and then incubated for 1 h with peroxidase conjugated secondary antibodies (1:10,000, Abcam). Finally, chemiluminescence detection was performed and at least repeated for three times.

### Tumorsphere Culture

After trypsinization to obtain a single-cell suspension, serum free medium was used to resuspend human Cal-62 thyroid cancer cells. Cells were seeded (5,000 cells/5,000 μl/well) in 24-well low-attachment plates with Serum free medium in triplicate. After treatment with medium, GSK-J4 or doxorubicin alone or a combination of GSK-J4 and doxorubicin (DOX: GSK-J4 = 0.156:0.078 μM), the number of spheres in each well was counted after 6 d of incubation.

### Tumor Xenograft Models

Female BALB/c nude mice (6–8 weeks old, weight > 18 g) were obtained from the animal core facility of Nanjing Medical University. Mice were raised under specific pathogen-free conditions according to protocols approved by the animal laboratory of Zhongshan School of Medicine. After 1 week of adaptation, mice were injected subcutaneously in the axillary region with 1.4×10^6^ Cal-62 cells in 200 μl of serum-free media. The mice implanted with tumor cells were randomly distributed into four groups (n = 3 per group) and received GSK-J4 alone, doxorubicin alone, a combination of GSK-J4 and doxorubicin, or vehicle (PBS) by intraperitoneal injection once daily at a dose of 0.25 ml/10 g body weight. The mice were treated with the above strategy continuously for 14 d, and tumor volume was recorded every 2 d by caliper measurement of tumor diameter and calculated according to the following formula: V = L × W^2^/2 (L, length; W, width). Fourteen days after treatment, the mice were sacrificed, and the tumors were resected and weighed. All animal experiments were conducted according to the “Guidelines for the Welfare of Animals in Experimental Neoplasia”. This study was carried out in accordance with the principles of the Basel Declaration and the recommendations of the ARRIVE (Animal Research: Reporting of In Vivo Experiments) guidelines for avoiding or reducing animal experiments and the suffering of laboratory animals. The animal research protocol was approved by the ethical committee of the First Affiliated Hospital of Sun Yat-sen University.

### Statistical Analyses

All the *in vitro* experiments were repeated at least three times. Continuous variables were represented as mean ± standard deviation (SD). The significance of differences between samples *in vitro* assays was determined by Student’s t-test. In animal experiments, two-way repeated measures analysis of variance (ANOVA) was used to compare the differences among groups. In all the statistical analyses, *p* < 0.05 is considered to be statistically significant.

## Results

### GSK-J4 Inhibits the Proliferation of Human ATC Cells

**Figure 1:**
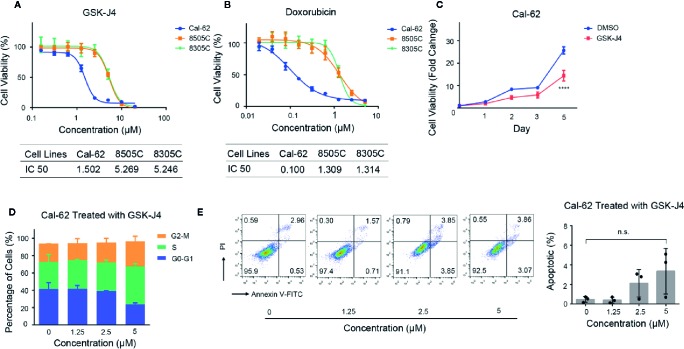
Effect of GSK-J4 on the Cal-62 Anaplastic Thyroid Carcinoma Cell Line. The proliferation relationship between concentration of GSK-J4 **(A)** and doxorubicin **(B)** in anaplastic thyroid cancer cell lines. **(C)** Cal-62 cell proliferation in different treatment time of GSK-J4. Effects of GSK-J4 on the cell cycle **(D)** and apoptosis **(E)** in Cal-62 cells. IC50, half maximal inhibitory concentration.

The antiproliferative effect of GSK-J4 and doxorubicin on ATC cells was measured by a cell viability assay. The data indicated that GSK-J4 efficiently inhibited the proliferation of ATC cells. After treatment for 48 h, the half maximal inhibitory concentrations (IC50s) of GSK-J4 in Cal-62, 8505C, and 8305C cells were 1.502, 5.269, and 5.246 μM, respectively (**Figure 1A**), and the IC50s of doxorubicin in Cal-62, 8505C, and 8305C cells were 0.100, 1.309, and 1.314 μM, respectively (**Figure 1B**). GSK-J4 had a continuing impact on Cal-62 cells over time (**Figure 1C**, *p* < 0.05). The results of the cell cycle analysis indicated that more ATC cells were blocked in G2-M and S phase with increasing drug concentrations (**Figure 1D**). These results suggest that GSK-J4 may cause cell damage, resulting in DNA replication being blocked. And the results of the apoptotic test showed that treatment with GSK-J4 induces cell apoptosis (**Figure 1E**, *p* < 0.05).

### The Combination of GSK-J4 and Doxorubicin Inhibits the Proliferation of Cal-62 Cells

**Figure 2:**
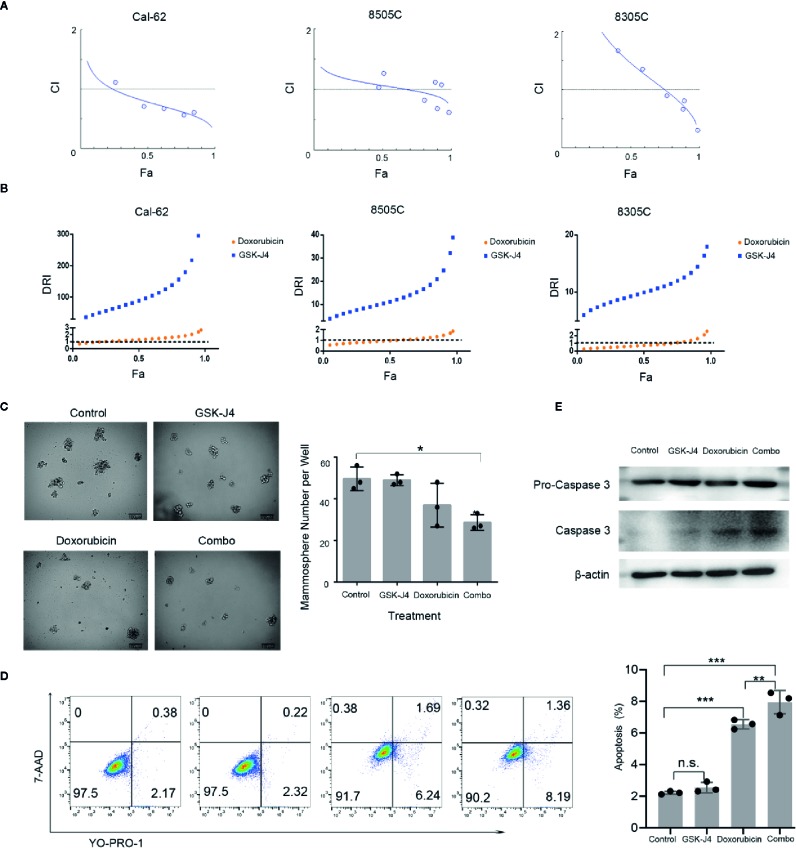
The Effect of GSK-J4 Combined With Doxorubicin. Synergistic curves of Cal-62, 8505C, and 8305C cell lines treated with GSK-J4 and doxorubicin **(A)** DRI of Cal-62, 8505C, and 8305C cell lines treated with GSK-J4 and doxorubicin **(B)** The effect of GSK-J4 combined with doxorubicin on the sphere-forming ability **(C)** of the Cal-62 cell line. Scale bar, 100 μM. YO-PRO-1/7-AAD staining show the effect of GSK-J4 combined with doxorubicin on cell apoptosis of the Cal-62 cell line **(D)**, Western blot showed the caspase 3 and Pro-caspase 3 with the treatment of effect of GSK-J4 combined with doxorubicin **(E)** (DOX: GSK-J4 = 0.156:0.078 μM). DRI, Dose reduction index. n.s., no statistical difference. *, p < 0.05, **, p < 0.01, ***, p < 0.001.

We treated ATC cell lines with the combination of GSK-J4 and doxorubicin, which showed a similar effect in 8505C and 8305C cells. With increases in the concentration of GSK-J4 and doxorubicin, the antagonistic effect first changed to a synergistic effect, but with continued increases in the concentration, the effect changed back to an antagonistic one. Thus, the two drugs exhibited synergy over a narrow concentration interval. The synergistic effect was slightly stronger in 8305C cells than in 8505C cells. When the two drugs acted on Cal-62 cells, the synergistic effect was obvious at lower concentrations than those seen in other cell lines, and the effect was stronger than that seen in the other two cell lines (**Figure 2A**). Computerized simulation of DRI indicated that at 75–97% growth inhibition levels, the doses of GSK-J4 could be reduced by 138.51-fold and 367.02-fold and the doses of doxorubicin by 1.63-fold and 2.67-fold in Cal-62 cells, the doses of GSK-J4 could be reduced by 16.66-fold and 38.90-fold and the doses of doxorubicin by 1.19-fold and 1.85-fold in 8505C cells, the doses of GSK-J4 could be reduced by 11.97-fold and 17.93-fold and the doses of doxorubicin by 1.09-fold and 2.58-fold in 8305C cells, respectively, when the drugs are used in combination (**Figure 2B**). We selected the ratio (DOX: GSK-J4 = 2:1) and drug concentration ratio (DOX: GSK-J4 = 0.156:0.078, Fa value 0.624, CI value 0.673) with the strongest synergistic effects to use for subsequent experiments (**Supplemental Tables 1–3**). Tumorsphere culture was performed in a KRAS-mutant cell line (Cal-62) to evaluate the suppressive abilities of the combination of GSK-J4 and doxorubicin. The combination of GSK-J4 and doxorubicin inhibited the 3D sphere growth of Cal-62 cells (**Figure 2C**). The number of tumorspheres per well treated with GSK-J4 alone, doxorubicin alone, and the combination was 49 ± 3, 37 ± 11, and 29 ± 4, respectively, while that of the control group was 50 ± 6 (**Figure 2C**). In addition, from the cell images, we can see that the average diameter of the tumorspheres treated with the combination was significantly smaller than that of the tumorspheres in the control group (**Figure 2C**). To explore the combination effect on apoptosis, we tested YO-PRO-1/7-AAD staining cell in different treatment groups and found apoptosis rate in the combined group increased significantly (**Figure 2D**). And combination of GSK-J4 and doxorubicin synergistically increased the caspase 3 level (**Figure 2E**). These data suggest that the combination of GSK-J4 and doxorubicin suppresses the sphere-forming abilities and growth of human ATC cells through inducing cellular apoptosis.

### The Combination of GSK-J4 and Doxorubicin Inhibits the Migration and Invasion of Cal-62 Cells

**Figure 3:**
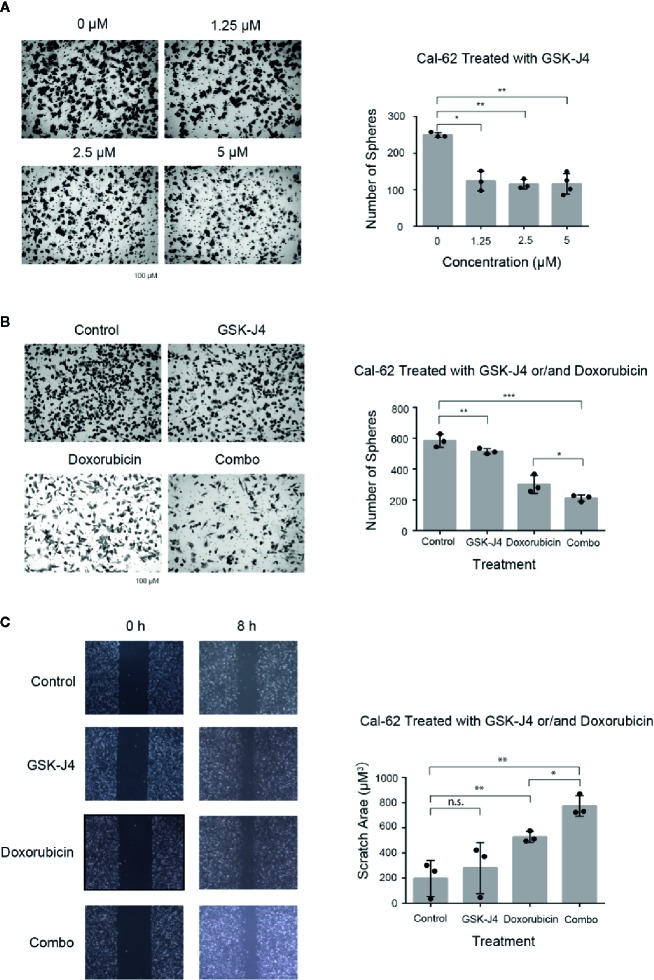
Effects of GSK-J4 and Doxorubicin on Invasion and Migration of the Cal-62 Cell Line. The invasion ability of GSK-J4 in different concentration on Cal-62 cell line **(A)** the effect of GSK-J4 combined with doxorubicin on the invasion ability **(B)** and migration ability **(C)** of the Cal-62 cell line. Scale bar, 100 μM. n.s., no statistical difference. *, p < 0.05, **, p < 0.01, ***, p < 0.001.

Transwell chamber assay results showed that the number of migratory cells was significantly (p < 0.05) reduced in Cal-62 cells that were treated with GSK-J4 when compared with the number in nontreated cells (**Figure 3A**). The number of cells that migrated per well in groups treated with 1.25, 2.5, or 5 μM GSK-J4 was 163 ± 10, 155 ± 9, and 158 ± 8, respectively, while that of the control group was 207 ± 11 (**Figure 3A**, *p* < 0.05). These data suggest that GSK-J4 inhibits migration in human thyroid cancer cells in a dose-dependent manner. In addition, when Cal-62 cells were treated with a single drug or a combination of both, the number of cells that migrated per well treated with GSK-J4, doxorubicin, or both was 515 ± 10, 312 ± 28, and 212 ± 12, respectively, while that of the control group was 584 ± 24 (**Figure 3B**, *p* < 0.05).

Scratch/wound-healing assays were performed in Cal-62 cell lines to evaluate the inhibitory effect of the combination of GSK-J4 and doxorubicin on tumor cell migration (**Figure 3C**). The data indicated that cell monolayer healing after 8 h was delayed in Cal-62 cells treated with a combination of GSK-J4 and doxorubicin when compared with nontreated cells and cells treated with a single drug alone (**Figure 3C**, *p* < 0.05).

### Treatment With a Combination of GSK-J4 and Doxorubicin Inhibits the Growth of Cal-62 Cell Xenografts in Nude Mice

**Figure 4:**
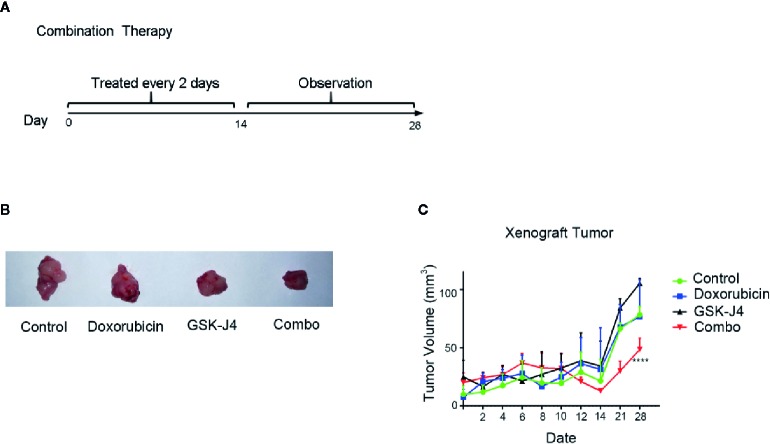
Effect of GSK-J4 combined with doxorubicin on the tumorigenic ability of Cal-62 cells. The schematic diagram of *in vivo* experiment **(A)** tumor tissues **(B)** and volume **(C)** treated by different experimental groups.

We investigated the antitumor effect of treatment with a combination of GSK-J4 and doxorubicin in nude mice bearing Cal-62 ATC xenografts. Intraperitoneal injection of a combination of GSK-J4 and doxorubicin every 2 d produced a significant sustained inhibitory effect (**Figure 4A**). The data showed that the growth of tumors in the groups treated with the combination of GSK-J4 and doxorubicin was significantly slower than that in the control group, GSK-J4 alone group, or doxorubicin alone group (**Figures 4B, C**). The inhibition rate was 38.0% in the groups treated with a combination of GSK-J4 and doxorubicin (*p* < 0.05). There were no obvious effects on the body weight of mice in the animal studies described above (data not shown), indicating that the combination of GSK-J4 and doxorubicin is likely well tolerated.

## Discussion

In this study, GSK-J4 significantly inhibited the proliferation of ATC cells, and the combination of GSK-J4 and doxorubicin had a stable synergistic effect in KRAS-mutant cell lines, which allowed for the inhibition of the sphere-forming abilities, tumorigenicity, migration, and invasion of the Cal-62 cell line at a low dose of doxorubicin.

Cal-62 cells have KRAS mutation and wild-type BRAF (KRAS G12R and BRAF wt, respectively). These alterations are common among anaplastic types of the disease, but few studies have been carried out. Additionally, 8505C and 8305C cells have BRAF gene mutations (Hoffmann et al., 2006; Sasanakietkul et al., 2018). Many new studies have been carried out on ATC patients with BRAF mutations, such as targeted therapy (Iyer et al., 2018; Knauf et al., 2018; Subbiah et al., 2018; ElMokh et al., 2019) and immunotherapy (Cabanillas et al., 2018). Although dabrafenib combined with trametinib has been widely accepted as an effective method for the treatment of BRAF mutation, there is no consensus that this therapy can be used for the treatment of KRAS mutant patients.

At present, the chemotherapeutic drugs are still one of the most important treatment for ATC, such as anthracyclines (doxorubicin) and paclitaxel (taxol and docetaxel) (Kouzarides, 2007; Kubicek et al., 2007; Helin and Dhanak, 2013). Moreover, it has been confirmed that the JAK-STAT signaling pathway is activated in RAS-positive ATC (Yoo et al., 2019). Aziz Zaanan et al. found that KRAS mutation increased the level of BCL-XL expression by elevating the level of STAT3 (signal transducer and activator of transcription 3) (Lin et al., 2013). Increased expression of BCL-XL was associated with decreased sensitivity of cells to chemotherapeutic drugs.

Because single drug cannot receive satisfactory results. The synergistic treatment of the two drugs is another option to improve the clinical efficacy. Several studies have reported that a combination of two or more drugs may benefit patients with anaplastic disease (Saini et al., 2018; Cao et al., 2019; Chintakuntlawar et al., 2019; Schurch et al., 2019). For example, combination sorafenib with quinacrine (Ha et al., 2017) and a combination of the BH3 mimic drug ABT-737 and doxorubicin (Ntziachristos et al., 2014) induced ATC cell apoptosis. Yong Sang Lee et al. tested primary cells cultured from ATC patients and found that different combinations of HNHA (a histone deacetylase), lenvatinib (a fibroblast growth factor receptor inhibitor), and sorafenib (a tyrosine kinase inhibitor) were more effective than single drugs (Hjelmeland et al., 2017). However, these discoveries are still in the basic experimental stages, and there are still many problems to be solved before their actual clinical application. Research related to clinical treatment has mainly focused on the efficacy of single-drug chemotherapy (Hu et al., 2012; Del Rizzo and Trievel, 2014; Ljubas et al., 2019), but these studies have not discussed the reasons for the poor efficacy of single drugs in treatment. The most effective drug, doxorubicin, has side effects, such that the dosage is strictly limited. In addition, some cancer cells easily resist doxorubicin. Even with doxorubicin chemotherapy, most patients still cannot avoid disease progression in the course of treatment. In cases in which existing single-drug therapies cannot effectively inhibit cancer progression, choosing two drugs with a synergistic effect to achieve a better therapeutic outcome than what would be achieved with a single drug while reducing the toxic side effects of the drugs may be another therapeutic option.

In the previous literature review, we knew that KRAS mutation upregulates the expression of STAT3. Maureen M. Sherry-Lynes et al. found that the transcription regulator STAT3 can directly bind to the JMJD3 promoter, specifically inducing JMJD3 expression and resulting in demethylation of H3K27me3, thereby affecting the expression of downstream genes that have been proven to be related to chemosensitivity. Ken Shiraiwa et al. suggested that STAT3 played an important role in ATC stem cells (Shiraiwa et al., 2019). After treatment with a STAT3 inhibitor, cancer cells were sensitized to chemotherapeutic drugs (Borbone et al., 2010). Thus, GSK-J4 is likely to block the effect of STAT3 on JMJD3 and play a similar role to STAT3 inhibitors and thus enhance the sensitivity of ATC to doxorubicin. In this study, we showed that combination of GSK-J4 and doxorubicin synergistically and significantly induces the apoptosis of human ATC cells through increasing caspase 3 level. The detailed molecular mechanism of synergistic therapy needs to be further studied.

**Figure 5:**
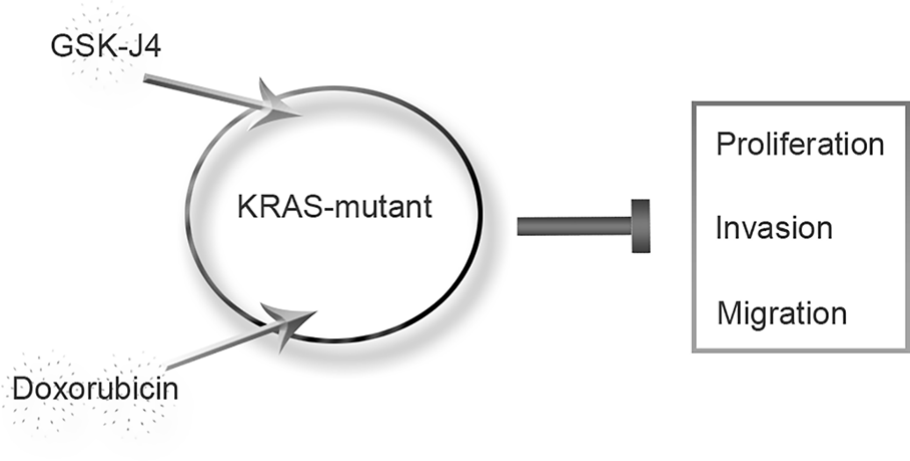
Proposed model of KRAS-mutant anaplastic thyroid cancer with GSK-J4 and doxorubicin treatment.

In conclusion, we found that the JMJD3 inhibitor GSK-J4 significantly inhibited the proliferation of ATC. In KRAS-mutant cells (the Cal-62 cell line), the synergistic effect of GSK-J4 and doxorubicin was obvious at lower concentrations, and the effect was stronger than that seen in the BRAF-mutant cell lines (**Figure 5**). Our findings provide a new method for the systemic treatment of KRAS-mutant ATC.

## Data Availability Statement

All datasets generated for this study are included in the article/**Supplementary Material**.

## Ethics Statement

The animal study was reviewed and approved by the animal laboratory of Zhongshan School of Medicine.

## Author Contributions

Equal contributors: BLi, I-YH, and BLu. Data collection and drafting: JL, WL, WZ, and BLi. *In vivo* experiments: I-YH and BLi. *In vitro* experiments: BLi and BLu. Statistical analysis: ZL and ZS. Manuscript polishing: BLi. Building figures: I-YH and BLu. Manuscript editing: JL and WL. Manuscript revision: BLi, BLu, YY and WZ. All authors contributed toward data analysis, drafting and critically revising the paper, gave final approval of the version to be published, and agree to be accountable for all aspects of the work.

## Funding

We acknowledge the support received from the Guangdong Provincial Science and Technology Department Research Projects (grant No. 2017A010105029 and grant No. 2016A040403049), National Natural Science Foundation of China (grant No. 81702784), Medical Scientific Research Foundation of Guangdong Province of China (grant No. A2017110), Special funds for Dapeng New District Industry Development (grant No. KY20160309) and Natural Science Foundation of Guangdong Province (grant No. 2017A030310228).

## Conflict of Interest

The authors declare that the research was conducted in the absence of any commercial or financial relationships that could be construed as a potential conflict of interest.
