# Supplementary material for: Synergy of GSK-J4 With Doxorubicin in KRAS-Mutant Anaplastic Thyroid Cancer
Source: Front Pharmacol. 2020 May 13;11:632. doi: 10.3389/fphar.2020.00632 (PMC7239034; doi:10.3389/fphar.2020.00632)
Supplement: Supplementary file 1 [file DataSheet_1.docx]

Supplementary Information

Synergy of GSK-J4 with Doxorubicin in KRAS-mutant Anaplastic Thyroid Cancer

Bo Lin, Bing Lu, I-yun Hsieh, Zhen Liang, Zicheng Sun, Weiming Lv, Wei Zhao, Jie Li.

**Contents**

**Table S1-S3**

**Table S1. Synergistic effects of GSK-J4 or/and Doxorubicin (DOX) in Cal-62 cells (DOX: GSK-J4=2:1).**

| ***Dose*** | ***A*** | ***B*** | ***C*** | ***D*** | ***E*** | ***F*** | ***G*** | ***H*** |
| --- | --- | --- | --- | --- | --- | --- | --- | --- |
| **DOX** | **22389** | **19245** | **11950** | **10131** | **5555** | **4370** | **4073** | **3314** |
|  | **21422** | **20433** | **9633** | **8799** | **6725** | **4352** | **3890** | **2883** |
| **GSK-J4** | **27317** | **27568** | **25683** | **23861** | **22857** | **23568** | **22880** | **9349** |
|  | **24254** | **24190** | **23499** | **23945** | **25721** | **22936** | **20221** | **11267** |
| ***Dose*** | ***a*** | ***b*** | ***c*** | ***d*** | ***e*** | ***f*** | ***g*** | ***h*** |

| ***Dose*** | ***Control*** | ***Aa*** | ***Bb*** | ***Cc*** | ***Dd*** | ***Ee*** | ***Ff*** | ***Gg*** | ***Hh*** |
| --- | --- | --- | --- | --- | --- | --- | --- | --- | --- |
| **DOX: GSK-J4=2:1** | **25033** | **21292** | **18273** | **11385** | **7421** | **4881** | **3552** | **3509** | **3063** |
|  | **23971** | **22733** | **18417** | **13116** | **9553** | **5689** | **3837** | **3348** | **3019** |
|  | **25670** | **22678** | **17625** | **13019** | **9185** | **5707** | **3989** | **3784** | **3219** |

| ***Dose**** | ***A*** | ***B*** | ***C*** | ***D*** | ***E*** | ***F*** | ***G*** | ***H*** |
| --- | --- | --- | --- | --- | --- | --- | --- | --- |
| **DOX** | **0.02** | **0.039** | **0.078** | **0.156** | **0.313** | **0.625** | **1.25** | **2.5** |
| **GSK-J4** | **0.01** | **0.02** | **0.039** | **0.078** | **0.156** | **0.313** | **0.625** | **1.25** |
| ***Dose*** | ***a*** | ***b*** | ***c*** | ***d*** | ***e*** | ***f*** | ***g*** | ***h*** |

*** μM**

**Table S2. Synergistic effects of GSK-J4 or/and Doxorubicin in 8505C cells**

**(DOX: GSK-J4=2:1).**

| ***Dose*** | ***A*** | ***B*** | ***C*** | ***D*** | ***E*** | ***F*** | ***G*** |
| --- | --- | --- | --- | --- | --- | --- | --- |
| **DOX** | **11664** | **11285** | **9342** | **3353** | **1201** | **1142** | **1710** |
|  | **11579** | **10871** | **9389** | **3170** | **1160** | **1149** | **1716** |
| **GSK-J4** | **14994** | **15329** | **15277** | **15570** | **14766** | **13300** | **7499** |
|  | **15065** | **15079** | **15855** | **15102** | **15655** | **14661** | **7251** |
| ***Dose*** | ***a*** | ***b*** | ***c*** | ***d*** | ***e*** | ***f*** | ***g*** |

| ***Dose**** | ***A*** | ***B*** | ***C*** | ***D*** | ***E*** | ***F*** | ***G*** |
| --- | --- | --- | --- | --- | --- | --- | --- |
| **DOX** | **0.156** | **0.313** | **0.625** | **1.25** | **2.5** | **5** | **10** |
| **GSK-J4** | **0.078** | **0.156** | **0.313** | **0.625** | **1.25** | **2.5** | **5** |
| ***Dose**** | ***a*** | ***b*** | ***c*** | ***d*** | ***e*** | ***f*** | ***g*** |

| ***Dose*** | ***Control*** | ***Aa*** | ***Bb*** | ***Cc*** | ***Dd*** | ***Ee*** | ***Ff*** | ***Gg*** |
| --- | --- | --- | --- | --- | --- | --- | --- | --- |
| **DOX: GSK-J4=2:1** | **14017** | **12087** | **11405** | **8013** | **3186** | **1349** | **1186** | **1235** |
|  | **14277** | **12630** | **12404** | **9295** | **4087** | **1712** | **1362** | **1285** |
|  | **13870** | **13439** | **11489** | **9962** | **4390** | **1439** | **1273** | **1247** |

*** μM**

**Table S3. Synergistic effects of GSK-J4 or/and Doxorubicin in 8305C cells**

**(DOX: GSK-J4=2:1).**

| ***Dose*** | ***A*** | ***B*** | ***C*** | ***D*** | ***E*** | ***F*** | ***G*** |
| --- | --- | --- | --- | --- | --- | --- | --- |
| **DOX** | **11505** | **9879** | **4928** | **2417** | **2110** | **2341** | **2765** |
|  | **11205** | **10075** | **5376** | **2277** | **1965** | **2328** | **2844** |
| **GSK-J4** | **13963** | **13044** | **13106** | **13355** | **13320** | **11926** | **6115** |
|  | **12959** | **13767** | **12850** | **13337** | **12998** | **10384** | **6162** |
| ***Dose*** | ***a*** | ***b*** | ***c*** | ***d*** | ***e*** | ***f*** | ***g*** |

| ***Dose**** | ***A*** | ***B*** | ***C*** | ***D*** | ***E*** | ***F*** | ***G*** |
| --- | --- | --- | --- | --- | --- | --- | --- |
| **DOX** | **0.156** | **0.313** | **0.625** | **1.25** | **2.5** | **5** | **10** |
| **GSK-J4** | **0.078** | **0.156** | **0.313** | **0.625** | **1.25** | **2.5** | **5** |
| ***Dose**** | ***a*** | ***b*** | ***c*** | ***d*** | ***e*** | ***f*** | ***g*** |

| ***Dose*** | ***Control*** | ***Aa*** | ***Bb*** | ***Cc*** | ***Dd*** | ***Ee*** | ***Ff*** | ***Gg*** |
| --- | --- | --- | --- | --- | --- | --- | --- | --- |
| **DOX: GSK-J4=2:1** | **15998** | **12167** | **11431** | **6695** | **2586** | **1901** | **2593** | **1841** |
|  | **14023** | **11851** | **10870** | **6307** | **2505** | **1807** | **2295** | **1929** |
|  | **13074** | **11749** | **9918** | **5908** | **2624** | **1836** | **1992** | **1571** |

*** μM**
